# Prognostic value of cranial ultrasound findings in infants aged <90 days with bacterial meningitis: a single-centre retrospective cohort study

**DOI:** 10.1136/bmjpo-2024-002691

**Published:** 2024-07-24

**Authors:** Ying Liu, Lili Liu, Rui Zhang, Zezhong Tang, Xinlin Hou

**Affiliations:** 1 Department of Neonatal Ward, Peking University First Hospital, Beijing, China; 2 Department of Pediatrics, Peking University Shenzhen Hospital, Shenzhen, China

**Keywords:** Child Health, Neurology

## Abstract

**Background:**

Bacterial meningitis (BM) poses a serious threat to infant health. We assessed cranial ultrasound (CUS) changes in infants with BM as possible predictors of the neurological sequelae of BM.

**Methods:**

We retrospectively assigned 132 infants diagnosed with BM from 2007 to 2021. Neuroimaging characteristics and cerebral blood flow (CBF) profiles identified using CUS were analysed and compared between the groups during the acute and postacute phases of BM.

**Results:**

Overall, 102 infants with CUS and outcome data were recruited. 37/102 (36.3%) infants with neurological developmental impairments comprised the group with sequelae. Abnormal CUS findings increased the risk of sequelae during the postacute phase compared with the acute phase of BM. Prolonged white matter hyperechogenicity was an independent risk factor for sequelae. The CBF profiles of the group with sequelae showed that anterior cerebral artery resistance and pulsatility indices decreased during the acute phase, whereas the mean flow velocity of the middle cerebral artery significantly increased during the postacute phase. Changes in the CBF profiles did not significantly differ in the group without sequelae.

**Conclusions:**

Serial CUS can facilitate the prognostic assessment of infants aged <90 days with BM. Prolonged white matter hyperechogenicity, brain volume loss and cerebral perfusion disorders contribute to the risk of sequelae.

**WHAT IS ALREADY KNOWN ON THIS TOPIC**

- Portable and non-invasive cranial ultrasound (CUS) can serve as a screening tool to evaluate the initial signs and complications of bacterial meningitis (BM). Some efforts have been made to explore the potential value of CUS in the determination of BM prognosis.

**WHAT THIS STUDY ADDS**

- CUS findings at different BM stages have different effects on prognosis. Abnormal CUS findings during the postacute phase were associated with an increased risk of sequelae compared with those during the acute phase. Prolonged white matter hyperechogenicity, brain volume loss and cerebral perfusion disorders contribute to the risk of sequelae.

**HOW THIS STUDY MIGHT AFFECT RESEARCH, PRACTICE, OR POLICY**

- Serial CUS can facilitate prognosis assessment in infants aged <90 days with BM.

## Introduction

 Bacterial meningitis (BM) threatens infant health, with high mortality and morbidity.^1 2^ In a previous study, the prevalence of BM in hospitalised infants aged 0–7 and 7–59 days was 3.0% and 4.8%, respectively.^3^ The mortality rate of neonatal BM is 10%–15%, with 20%–58% of survivors developing neurodevelopmental sequelae.^4^,^6^ To improve prognoses, identifying predictors of neurological sequelae early is required for effective treatment, follow-up and interventions. Portable and non-invasive cranial ultrasound (CUS) can be an effective screening tool for the initial signs and complications of BM.^7^ However, few studies have explored the prognostic factors of BM. Therefore, this study aimed to determine changes in CUS findings in infants aged <90 days with BM and to identify predictors of neurological sequelae to improve disease detection in clinical practice and research.

## Materials and methods

### Study participants

This retrospective single-centre cohort study identified 132 infants diagnosed with BM (onset age ≤90 days) at Peking University First Hospital from 2007 to 2021. The Biomedical Research Ethics Committee at Peking University First Hospital approved this study (approval ID: 2022-YAN-299-003). This study adhered to the Declaration of Helsinki (2013 amendment) principles. Exemption from the ethics committee was obtained mostly for informed consent, and a few participants who were less than 18 months of age at the time of their initial inclusion were informed of the relevant content by telephone with their parents or legal guardians. The enrollment criteria were to meet the following: (1) temperature instabilities, lethargy, poor feeding, vomiting and convulsions; (2) bulging anterior fontanelle, abnormal muscle tone and positive meningeal irritation sign; (3) cerebrospinal fluid (CSF) white blood cell count (WBC) >20 ×10^6^/L, multinucleated cell ratio >0.6, increased total protein (>1.7 g/L for neonates and >0.4 g/L for infants aged 29–89 days) and decreased blood glucose (<2.2 mmol/L or CSF glucose/blood glucose ratio <0.4); and (4) positive bacteria in CSF smears or cultures. The exclusion criteria comprised neurodevelopmental malformations (such as meningocele), genetic metabolic diseases, severe hypoxic-ischaemic encephalopathy, bilirubin encephalopathy, trauma, infants with gestational ages <28 weeks or birth weight <1000 g and/or death or loss of follow-up.

### Methods

This study is a retrospective cohort study. The infants with CUS records and follow-up data for at least 18 months were eventually included in the analysis. During the follow-up phase, certified examiners evaluated the infants using the Bayley Scales of Infant and Toddler Development III. Sequelae were defined as having a consequent neurological developmental impairment (NDI) with at least one of the following: (1) Bayley III cognitive, language or motor composite scores <70^8^; (2) definitive cerebral palsy with any level of Gross Motor Function Classification System^9^; and (3) sensorineural or mixed hearing loss or unilateral or bilateral visual impairment. All CUS procedures were conducted by experienced sonographers according to neurosonography guidelines for infants.^10 11^ The sonographic neuroimaging findings and cerebral blood flow (CBF) profiles of the middle cerebral artery (MCA) and anterior cerebral artery (ACA) on transcranial Doppler ultrasound (TCD), such as mean flow velocity (Vm), resistance index (RI=(Vsystolic−Vdiastolic)/Vsystolic), pulsatility index (PI=(Vsystolic−Vdiastolic)/Vm) and ratios of systolic and diastolic flow velocity (S/D), were analysed. Considering temporal heterogeneity and differences in CUS findings between the early and late stages of BM, we stratified the infants according to whether CUS was performed within (acute) or after 7 days (postacute) of onset. General characteristics and CUS findings from medical records were compared between groups.

### Patient and public involvement

Patients or the public were not involved in the design, conduct, reporting, or dissemination plans of our research.

### Statistical analysis

Data were analysed using SPSS (V.19; IBM Corp, Armonk, New York, USA). Normally distributed continuous variables are presented as mean±SD and were compared using the Student’s t-test. Non-normally distributed variables are presented as medians with IQR and were compared using the Wilcoxon rank-sum test. Categorical variables are presented as frequencies (%) and were compared using the Pearson χ^2^ or Fisher’s exact test. Relationships between CUS characteristics and BM sequelae were analysed using multivariate logistic regression. The p values <0.05 were considered statistically significant.

## Results

### General characteristics

**Figure 1:**
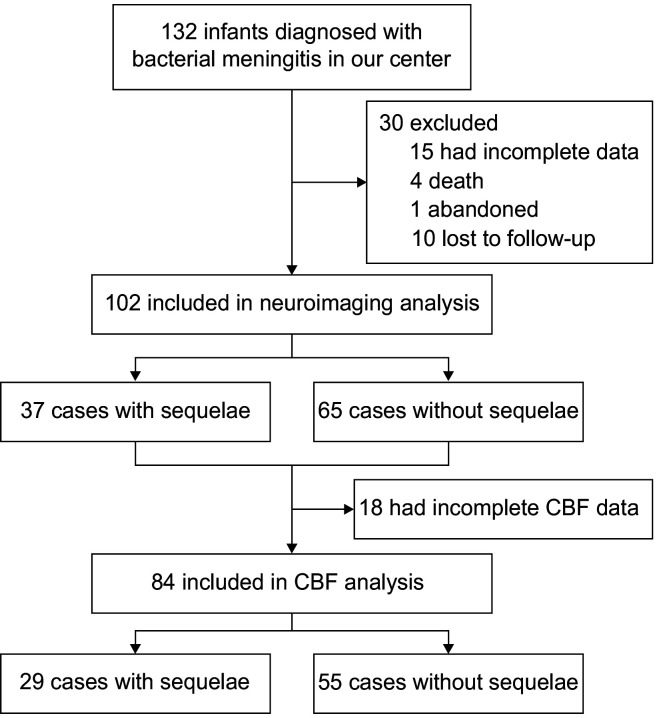
Flow diagram of study participants. CBF, cerebral blood flow.

Of 132 infants with BM, we excluded some due to death (n=4), abandonment (n=1), loss to follow-up (n=10) and incomplete data (n=15). Finally, 102 infants with CUS neuroimaging data and 84 infants with CBF profiles were included (figure 1). Overall, 37/102 (36.3%) infants with NDI comprised the group with sequelae. The interval between CUS and BM onset was significantly longer in the sequelae group than in the group without (p<0.05). The baseline characteristics of the patients are summarised in table 1.

**Table 1:** Characteristics of infants with bacterial meningitis

Characteristics	Sequelae(n=37)	No sequelae(n=65)	P value
Maternal age (years)	31.9±4.9	30.9±4.1	0.29
Maternal gestational diabetes	7 (10.8)	8 (21.6)	0.16
Gestational hypertension	2 (3.1)	4 (10.8)	0.19
Vaginal delivery, n (%)	42 (64.6)	20 (54.1)	0.30
Median gestational age, weeks	0.16	39 (36.3‒40)	0.16
Median birth weight, g	3000 (1915‒3410)	3180 (2650‒3430)	0.20
Male, n (%)	24 (64.9)	44 (67.7)	0.83
Asphyxia, n (%)	7 (10.8)	2 (5.4)	0.48
Median age at onset (days)	9 (3‒6)	6 (1‒24)	0.13
Cranial ultrasound after onset (d)	15 (6.5‒20.5)	6 (4‒12)	0.002

Data are shown as means±SD, n%, or medians (IQR)

### Correlations between neuroimaging findings and BM sequelae

84 (82.4%) of 102 infants had neuroimaging abnormalities on CUS. The rates of CUS abnormalities (%) were significantly higher in the group with sequelae than in those without (37 (100%) of 37 vs 47 (72.3%) of 65, p<0.05). Because there was a significant difference between the time of the first CUS, a stratified analysis of subgroups according to the time of the CUS was performed as the acute phase (within 1 week) and the postacute phase (≥1 week). The CUS of 45 infants was performed during the acute phase of BM at a mean of 4.5 (3‒6) days after onset. The rates of CUS abnormalities did not significantly differ between the groups with and without sequelae (p>0.05). CUS findings such as meningeal thickening, echogenic sulci, white matter hyperechogenicity (WMHE), changes in ventriculitis, intraventricular haemorrhage (IVH) and ventricular dilation were more likely to be found in the group with sequelae (all p<0.05; table 2). No independent risk factors for predicting neurological sequelae were identified by multivariate logistic regression analysis.

**Table 2:** Comparison of neuroimaging findings in infants with bacterial meningitis based on prognosis

	Acute phase (within 1 week)(n=45)					Postacute phase (≥1 week)(n=57)			
	N (%)		Sequelae(n=9)	No sequelae (n=36)	P	N (%)	Sequelae (n=28)	No sequelae (n=29)	P value
Meningeal thickening	21 (20.5)	5 (11.1)	4	1	0.004	16 (28.1)	9	7	0.55
Echogenic sulci	30 (29.4)	11 (24.4)	6	5	0.003	19 (33.3)	11	8	0.03
White matter hyperechogenicity*	50 (49.0)	20 (44.4)	5	15	0.48	30 (52.6)	20	10	0.02
Abnormal basal segments†	31 (30.4)	7 (15.6)	3	4	0.13	24 (42.1)	14	10	0.29
Malacia lesion	11 (10.8)	2 (0.4)	2	0	0.04	9 (15.8)	8	1	<0.001
Ventriculitis‡	37 (36.3)	8 (1.8)	4	4	0.04	29 (50.9)	16	13	0.03
Intraventricular haemorrhage	39 (38.2)	13 (28.9)	7	6	0.001	26 (45.6)	13	13	0.99
Ventricular dilation	41 (40.2)	7 (15.6)	5	2	0.002	34 (59.6)	19	15	0.28
Extra-axial fluid space enlargement	18 (17.6)	5 (11.1)	1	4	0.99	13 (22.8)	8	5	0.01
Infants with cranial ultrasound abnormalities	84 (82.4)	33 (73.3)	9	24	0.09	51 (89.5)	28	23	0.02

* Including increased or heterogeneous white matter echogenicity.

† Basal segment size reduction, extensive elevated echogenicity, focal elevated echogenicity, heterogeneous echogenicity or local vascularisation.

‡ Increased ventricular lining echogenicity, cerebrospinal fluid hyperechogenicity in the ventricles, echogenic debris within the ventricular cavity or dilation and hyperechogenicity of the choroid plexuses.

The CUS of 57 infants was performed during the postacute phase of BM at a mean of 16 (11–21) days after onset. The rates of CUS abnormalities were significantly higher in the group with than without sequelae (100% vs 79.3%; p<0.05). WMHE, malacic lesions and extra-axial fluid space (EAFS) enlargement were more often evident in the group with sequelae (table 2). WMHE (OR 2.205; 95% CI 1.737 to 47.371; p<0.05) seemed to predict neurological sequelae independently.

Overall, sequelae were more common when CUS abnormalities were found during the postacute phase than in the acute phase (28 (54.9%) of 51 vs 9 (27.3%) of 33; p<0.05).

### Correlations between CBF patterns and BM sequelae

We recorded the CBF profiles of 84 infants after hospital admission. The ACA-RI and ACA-PI significantly decreased (p<0.05) in the acute phase, and the MCA-Vm significantly increased in the postacute phase (p<0.05) in the group with sequelae (table 3). However, no independent predictors were identified (p>0.05 for both groups).

**Table 3:** Cerebral blood flow profiles of infants with BM according to prognosis

	Acute phase (within 1 week, n=43)			Postacute phase (≥1 week, n=41)		
	Sequelae(n=10)	No sequelae(n=33)	P	Sequelae(n=19)	No sequelae(n=22)	P value
Age (days)	27 (5–47)	16 (3–30)	0.41	44 (28–70)	36 (16–58)	0.23
Days after onset	4.00±1.76	4.64±1.52	0.27	18.11±5.57	13.41±6.05	0.01
ACA-Vm, cm/s	23.92±9.31	23.72±10.82	0.96	30.34±10.57	26.25±9.51	0.23
ACA-S/D	3.28 (2.68–4.01)	3.62 (3.19–4.70)	0.05	3.80 (3.10–5.18)	4.00 (3.63–5.01)	0.80
ACA-RI	0.70±0.07	0.74±0.07	0.05	0.75±0.08	0.75±0.06	0.86
ACA-PI	1.22±0.19	1.47±0.35	0.04	1.44±0.32	1.48±0.27	0.69
MCA-Vm, cm/s	36.77±8.25	29.95±7.71	0.07	45.50±15.14	29.47±7.73	0.02
MCA-S/D	3.39 (2.96–6.28	4.28 (3.29–5.20)	0.52	4.13 (3.43–5.05)	3.90 (3.42–4.47)	0.90
MCA-RI	0.73±0.10	0.75±0.08	0.56	0.76±0.06	0.75±0.05	0.64
MCA-PI	1.41±0.38	1.57±0.40	0.40	1.60±0.34	1.46±0.34	0.35

Data are shown as means±SD or medians with IQRs.

* Days (n) after onset of BM when infants were assessed by cerebral ultrasound.

ACAanterior cerebral arteryBMbacterial meningitisCUScerebral ultrasoundddaysIQRinterquartile rangeMCAmiddle cerebral arteryPIpulsatility indexRIresistance indexS/Dratio of maximum systolic and diastolic blood flow velocitiesVmmean flow velocity

**Figure 2:**
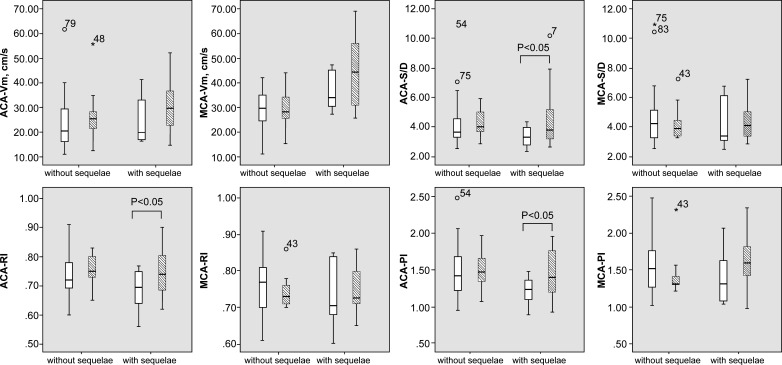
Changes in CBF profile from the acute to postacute stage in the groups with and without sequelae. Without (□) and with (▪) sequelae. ACA, anterior cerebral artery; CBF, cerebral blood flow; MCA, middle cerebral artery; PI, pulsatility index; RI, resistance index; S/D, ratio of maximum systolic and diastolic blood flow velocities; Vm, mean flow velocity.

A comparison of the evolution of blood flow parameters in infants from the acute to the postacute stage revealed that the ACA-S/D, ACA-RI and MCA-PI significantly increased (p<0.05), whereas the ACA-Vm and MCA-Vm increased (p>0.05) in the group with sequelae. None of the parameters significantly differed between the acute and postacute stages in the group without sequelae (figure 2).

## Discussion

We investigated changes in CUS and predictors of neurological sequelae among infants aged <90 days with BM. Abnormal CUS findings are evident in approximately 65% of patients during the acute phase of BM^7 12^ and in up to 100% of patients with severe neurological symptoms.^13^ We found that the rates of CUS abnormalities were 82.4% for all infants and 100% for the group with sequelae. Moreover, sequelae were more common when CUS abnormalities were found during the postacute phase than in the acute phase. These findings suggest that infants with CUS abnormalities during the postacute phase of BM are at increased risk of sequelae. Meningeal thickening and echogenic sulci that are prevalent and the earliest findings of BM^14^,^16^ were detected in 20.5% and 29.4% of our patients, respectively. A widening hyperechoic line on the surface of the cerebral gyri on sonograms due to inflammatory exudates accumulating around the pia and arachnoid spaces is a direct indication of arachnoiditis and/or subependymal gliosis.^17^ In our study, meningeal thickening was statistically different only in the acute phase, which was mostly seen in the sequelae group. With very few cases with this sign in the acute phase, the result needs to be explored further. Echogenic sulci are caused by brain oedema and appear with increased sulcal thickness and echogenicity. Advanced brain oedema presents as obfuscation of the boundaries between the gyri, as well as compression and deformation of the ventricular system and remaining fluid cavities.^16^ Echogenic sulci were significantly more frequent in the group with sequelae than in the group without. These findings suggest that a powerful inflammatory response with excessive exudate indicates an unfavourable prognosis.

Identifying such abnormalities is challenging, relying on the sonographer’s experience and the timing of the CUS. Despite their prevalence and value in assessing BM, these signs are temporary and unrelated to neurological outcomes.^7 18^ Consistently, we found that neither meningeal thickening nor echogenic sulci independently predicted neurological sequelae.

The most common brain parenchymal findings were WMHE, abnormal basal segments and malacic lesions. Parenchymal changes on sonography are diverse due to various pathological changes or different disease processes. They can present as areas of increased echogenicity, diffuse or focal infarction, abscess formation or heterogeneous malacic lesions with liquefied spaces as the disease advances.^7 19^ Three infants had infarction foci resulting from thrombosis secondary to vasculitis. These were initially visible as areas of heterogeneous echogenicity, with evident obfuscation of the echostructure that gradually developed into leukomalacic lesions in the affected area. Malacia lesions were more frequent in the group with sequelae both in the acute and postacute phase (p<0.05), and total sequelae developed in 10 (90.9%) of the 11 infants with leukomalacic lesions. This suggests that brain parenchymal loss indicates poor outcomes. One study of 19 neonates with Group B streptococcal meningitis during follow-up found increased echogenicity in sonographic images of the white matter, cortex, basal ganglia/thalamus and diffuse echogenicity in the cortex were associated with adverse composite motor outcomes.^7^ In our study, WMHE during the postacute phase at 16 (IQR 11‒21) days after BM onset predicted neurological sequelae independently. This suggested that infants with BM and prolonged WMHE were prone to poor neurological outcomes. A classification of brief, intermediate or prolonged hyperechogenicity as 1–6, 7–13 and ≥14 days, respectively, has been proposed.^20^ Previous studies of preterm infants have drawn similar conclusions^21 22^: the duration of periventricular hyperechogenicity correlates with poor long-term outcomes. Although WMHE is subjective, its accuracy as a predictor of sequelae requires further exploration.

Ventriculitis is detected by CUS as an irregular thickening and hyperechogenicity of the ventricular lining and/or intraventricular debris. Ventriculitis in 15.3%–20.8% of neonatal BM has resulted in mortality rates of 22.2%–33%.^23^,^25^ Ventriculitis developed in 36.3% of our infants, among whom 3 (8.1%) died and 20 (54.1%) developed neurological sequelae. At the same time, ventriculitis was more commonly found in the group with sequelae than without in both the acute and postacute phases (p<0.05). However, IVH can overlap with ventriculitis on CUS images, which might explain why it is not an independent risk factor for sequelae. Ventricular dilation has been identified in 14%–65% of infants with BM.^7 16^ The high incidence (40.2%) of ventricular dilation in this study might have been associated with disease severity and the fact that smaller infants are prone to hydrocephalus. We found NDI in 24 (58.5%) patients with hydrocephalus. Moreover, hydrocephalus was not an independent risk factor for sequelae, perhaps because active and effective treatment might have improved the prognosis. IVH and ventricular dilation were more frequent in the sequelae group only in the acute phase, suggesting early ventricular dilatation might indicate a rapid progression or large IVH that may be associated with poor outcomes.

We also detected EAFS enlargement in the subdural and/or subarachnoid space of 18 (17.6%) infants, which was statistically different only in the postacute phase possible because it is generally not easily seen in the early stage of BM and remains present in the later stage might tend to poor prognosis. Hygroma was diagnosed in eight of them. Hygromas containing sterile fluid present as homogeneously anechoic areas and usually do not alter prognoses, as <5% became infected in this study. That may be the reason why it is not an independent risk factor for sequelae.

TCD is a valuable tool for monitoring changes in CBF profiles. We identified clear differences in CBF profiles according to neurological outcomes. CBF velocity (CBFV) significantly increased throughout the acute course of BM (especially between the first 3–5 days from onset), then declined gradually with disease recovery. Up to 3 weeks might pass before the CBFV returns to normal.^26 27^ One study found that CBFV significantly increased in 50 infants (mean age 45 days) during the acute phase of BM compared with that in healthy controls and persisted for 3 months (p<0.01).^28^ Meanwhile, 14 of 33 patients had neurological sequelae after 1 year of follow-up, with a significantly lower mean CBFV (19.6±8.7 vs 34.5±13.0 cm/s, p<0.01). The mean CBFV was significantly lower in children with BM with sequelae than in those without.^29^ The authors concluded that CBF profiles might be early predictors of the prognosis of BM.

Two of our patients with obvious hypoperfusion had poor outcomes. One died, whereas the other developed severe hydrocephalus and ventriculitis with recurrent convulsions and was lost to follow-up. However, the CBFV did not significantly differ between the groups with and without sequelae during the acute period, indicating a general increase in CBFV due to inflammation. The CBFV stabilised after treatment during the postacute stage of BM and was significantly higher in the group with sequelae. This suggested that a persistent, powerful inflammatory reaction led to secondary ischaemic injury, resulting in a poor prognosis. However, the prognostic value of perfusion indices remains largely unexplored, and the results of all relevant published studies are inconsistent due to small samples, different ages, CUS durations and disease severity. Moreover, CBFV might be associated with total blood perfusion, vessel diameter and cerebrovascular resistance. Owing to this complexity, other parameters should be captured together with CBFV.

Ischaemic stroke can occur in 20%–25% of patients with BM,^30^,^32^ and the main pathological change on angiography is cerebral artery stenosis.^31^ A significantly increased CBFV of either basilar artery, especially if asymmetrical, might indicate arterial stenosis.^33^ Although the aetiology of stenosis is not yet fully understood, vasospasm, intra-arterial thrombosis, vasculitis and vessel compression by subarachnoid exudation are implicated. A significant increase in the CBFV due to arterial stenosis was more pronounced in the MCA than in the ACA because of the lack of collateral vessels in the MCA.

The resistance-related parameters PI, RI and S/D, which represent vascular autoregulation, were not influenced by the angle of insonation, with low interobserver variability. Moreover, impaired autoregulation correlates with poor prognoses.^34 35^ We found that the mean CBFV in infants with sequelae was slightly higher and accompanied by significantly decreased ACA-RI and ACA-PI during the acute phase of BM. Therefore, the high CBFV/low resistance mode might play a role in predicting BM outcomes. Furthermore, high flow/low PI or low flow are prone to poor outcomes in children with BM (82% vs 38%, p=0.001).^36^ A high CBFV combined with reduced RI indicates elevated Vd (Vdiastolic), which parallels the aggravation of cerebral oedema caused by inflammation. The Vd gradually returned to normal as oedema diminished, and slower Vd recovery indicated a worse clinical condition. In small infants, immature cerebrovascular autoregulation hampers the maintenance of normal oxygenation, nutrient supply and metabolic waste removal in the brain, leading to brain damage. Furthermore, a comparison of the acute and postacute evolution of CBF profiles might be meaningful. The CBF profiles did not significantly differ as BM progressed in infants without sequelae in this study. However, a significantly increased trend of several resistance parameters combined with a relatively higher CBFV in the group with sequelae suggested that a decline in vascular regulation ability correlates with a poor long-term prognosis.

This study has some limitations, including its relatively small sample and the use of two types of CUS machines. Future prospective studies should be performed to assess whether sonographic findings can improve the prediction of outcomes in infants.

In conclusion, careful serial CUS imaging and monitoring of cerebral haemodynamic changes during the progression of BM are helpful with early adverse prognostic warnings. Prolonged WMHE and loss of brain volume are predictors of BM sequelae. Cerebral perfusion disorders, such as persistent hyperperfusion, marked hypoperfusion and a decline in CBF regulation capacity, contribute to the neurological sequelae of BM. More investigations of novel CUS indicators, including sonographic images and flow profiles, are needed to identify more prognostic predictors.

## Data Availability

Data are available upon reasonable request.
