# Supplementary material for: Prognostic value of cranial ultrasound findings in infants aged <90 days with bacterial meningitis: a single-centre retrospective cohort study
Source: BMJ Paediatr Open. 2024 Jul 24;8(1):e002691. doi: 10.1136/bmjpo-2024-002691 (PMC11284905; doi:10.1136/bmjpo-2024-002691)
Supplement: Supplementary file 1 [file bmjpo-2024-002691.reviewer_comments.pdf]

PEER REVIEW HISTORY

BMJ Paediatrics Open publishes all reviews undertaken for accepted manuscripts. Reviewers are asked to complete a checklist review form and are provided with free text boxes to elaborate on their assessment. These free text comments are reproduced below.

ARTICLE DETAILS

**TITLE (PROVISIONAL)**	Prognostic value of cranial ultrasound findings in infants aged <90 days with bacterial meningitis: a single-center retrospective cohort study
**AUTHORS**	Ying, Liu<br>Lili, Liu<br>Rui, Zhang<br>Zezhong, Tang<br>Hou, Xinlin

VERSION 1 – REVIEW

**REVIEWER**	Dr. Ruchi Rai<br>Postgraduate Institute of Child Health<br>Sector 30, Noida<br>Noida<br>201303<br>India
**REVIEW RETURNED**	30-May-2024

**GENERAL COMMENTS**	I will like to congratulate the authors for their meticulous study which has important clinical implications. My comments after going through the manuscript thoroughly are:<br>1. Page 1, line 7, methods section: “we retrospectively assigned………with or without sequelae” the sequelae must have been diagnosed at 18 m when the final neurological assessment was done. Is it correct to state that this assignment was done retrospectively.<br>2. Material and methods: at what point of time the subjects were enrolled? Was it 18 m when the presence or absence of sequelae was confirmed? or was the enrollment done at time of diagnosis of bacterial meningitis?<br>3. Similarly, for informed consent, when was the consent taken?<br>4. The enrollment criteria (inclusion criteria) should have more clarity. Many criteria have been mentioned. Whether the presence of any one of the criteria or all of them was necessary for inclusion.<br>5. Positive meningeal irritation signs are not seen in young infants. (page 4, line 15)<br>6. Cut-off values for CSF protein and sugar should be mentioned, instead of mentioning “increased protein or decreased sugar”.<br>7. The way methodology has been described, it looks like babies were followed up prospectively for the study.<br>8. It is mentioned in the methods that the examiners were blinded to clinical variables. (page 4, line 27) Retrospective study does not need blinding.<br>9. Page 4, line 29 “developmental impairment (NDI) with ≤ 1 of the following…..”. Please explain ≤ 1 of the following. Is it any one of the following?<br>10. Page 4, line 30-31, “gross motor function classification system level ≥ 1”. Does it mean any level of GMFCS?<br>11. Page 5, general characteristics section: If the children were

	enrolled at 18 m when the final diagnosis of sequelae was made, then keeping death and lost to follow-up as exclusion criteria will not be appropriate.<br>12. Table 1: there was a significant difference between the time the first USG was done from the onset of the disease in both the groups. Is there an explanation for this significant difference in the timing?<br>13. Table 2: in the row of meningeal thickening, the total N (%) should be 21 (20.5) instead of 14 (13.7).<br>14. Although overall the acute abnormal CUS findings are not significantly different but individual findings like meningeal thickening, echogenic sulci, malacia lesion, IVH, ventricular dilatation were significantly different in the groups. This point can be highlighted in discussion or result section.<br>15. Table 2: It is noticed that there are some findings on CUS done in the acute phase are significantly seen more in the sequelae group. On the other hand, a different set of findings in the post-acute CUS are associated with sequelae. This point should also be discussed.

**REVIEWER**	Dr. Jaya Shankar Kaushik<br>All India Institute of Medical Sciences - Guwahati<br>Pediatrics<br>Department of Pediatrics, All India Institute of Medical Sciences, Guwahati, Assam, India<br>Guwahati<br>Assam<br>781101<br>India
**REVIEW RETURNED**	03-Jun-2024

**GENERAL COMMENTS**	It is a very interesting study. Few observations:<br>1. the Best way to address this research question would have been a prospective cohort design where the sonographic findings were followed clinically for 1 year to determine the neurological outcome. The current retrospective chart review study design is an inferior design to address this question. Was it a retrospective chart review of medical records or a retrospective cohort? If it was a retrospective cohort, what was the age of children at the time of enrolment?<br>2. I believe only those who had BM in infancy were enrolled. However, there might be a variation in the age at presentation. I can see that infants were followed up till 18 months of age. In that case, BM should have occurred by 12 months of age. Hence, there will be a lot of variation in the BM timing and follow-up duration. Were only cases of neonatal meningitis enrolled? Authors may clarify the methodology to avoid this confusion in readers' minds.<br>3. The timing of the CUS needs clarification. Was it done within 7 days? Is there any timing with the lumbar puncture?<br>4. Was the developmental assessment done at 18 months of age? A single cut-off DQ<70 might be inappropriate to classify as sequelae. A reference to the same can be provided.<br>5. Inclusion criteria mention raised CSF protein and low sugar without objectivity.<br>6. Were all children subjected to MRI as malformations are one of

	the exclusion criteria<br>7. Authors conclude that "careful CUS and monitoring of cerebral hemodynamics will prevent further cerebral insult" The statement may be reframed appropriately

VERSION 1 – AUTHOR RESPONSE

Dear Prof. Piyush Guptar,

Thank you for reviewing our manuscript and for the constructive comments, which greatly helped us to improve the manuscript. We have revised the manuscript in accordance with the comments. And point-by-point responses to the comments were provided below.

We hope that the revision is acceptable, and your favorable consideration of our manuscript is greatly appreciated. Best regards!

1. Page 1, line 7, methods section: "we retrospectively assigned.........with or without sequelae" the sequelae must have been diagnosed at 18 m when the final neurological assessment was done. Is it correct to state that this assignment was done retrospectively.

This study is a retrospective cohort study designed in 2023, Given the need for a minimum of 18 months of follow-up, the infants included were hospitalized before 31 December 2021 to ensure adequate follow-up time and outcome data available. To prevent ambiguity, I modified the description of the summary section accordingly:"Methods: We retrospectively assigned 132 infants diagnosed with BM from 2007 to 2021. .... (Page 2,Line 7)

Results: Overall, 102 infants with CUS and outcome data were recruited. 37/102 (36.3%) infants with neurological developmental impairments comprised the group with sequelae. 82.4%(84/102) of all infants had CUS neuroimaging abnormalities......" (Page 2,Line 11)

2. Material and methods: at what point of time the subjects were enrolled? Was it 18 m when the presence or absence of sequelae was confirmed? or was the enrollment done at time of diagnosis of bacterial meningitis?

The time of enrollment was when the infant was diagnosed with BM, and these infants were followed up regularly in our hospital after discharge. We judged it based on the outpatient information or the diagnostic information of Medical records at the time of a possible re-hospitalization due to illness. The infants at ⩾18 months (rather than 18 months of age) with neurodevelopmental abnormalities were included in the sequelae group.

Our center is good at the diagnosis and treatment of children's neurological diseases, with relatively complete diagnosis，treatment and follow-up system, as well as skilled medical staff. Thus, the feasibility and credibility of the information can be guaranteed

3. Similarly, for informed consent, when was the consent taken?

This study was conducted through retrospective analysis of medical records. Ethics approval was passed, mostly exemption from informed consent. A few infants lack of information were informed the relevant content of the study by telephone to obtain informed consent from the parents, which was also reflected in the ethical approval.

4. The enrollment criteria (inclusion criteria) should have more clarity. Many criteria have been mentioned. Whether the presence of any one of the criteria or all of them was necessary for inclusion.

For a more accurate expression, it was modified to read as following:

"The enrollment criteria were Meet the following ① - ③ or ④: ①temperature instabilities, lethargy, poor feeding, vomiting, convulsions, ②bulging anterior fontanelle, abnormal muscle tone，

positive meningeal irritation sign; ③cerebrospinal fluid (CSF) white blood cell count (WBC) >20 × 106/L, multinucleated cell ratio >0.6, increased total protein(>1.7 g/L), and decreased blood glucose(<2.2mmol/L or CSF glucose/blood glucose ratio <0.4); ④ positive bacteria in

CSF smears or cultures."

5. Positive meningeal irritation signs are not seen in young infants. (page 4, line 15)

Neck rigidity is regarded as a sign of meningeal irritation , which can be present in young infants.

6. Cut-off values for CSF protein and sugar should be mentioned, instead of mentioning "increased protein or decreased sugar"

We regarded increased total protein as (>1.7 g/L for neonate and >0.4 g/L for infant aged 29~89 days), and decreased blood glucose as (<2.2mmol/L or CSF glucose/blood glucose ratio <0.4)

7. The way methodology has been described, it looks like babies were followed up prospectively for the study.

8. It is mentioned in the methods that the examiners were blinded to clinical variables. (page 4, line 27) Retrospective study does not need blinding.

All infants with BM evaluated in our center are subjected to follow-up schedules， as they are considered to have a high risk in terms of neurodevelopmental problems. Professional physicians provide a thorough evaluation During the follow-up phase.For a more accurate expression, it was modified to read as following:

"This study conducted a retrospective analysis .The infants with CUS records and follow-up data for least 18 months were eventually included in the analysis. During the follow-up phase, certified examiners evaluated the infants using the Bayley Scales of Infant and Toddler Development III."

9. Page 4, line 29 "developmental impairment (NDI) with ≤ 1 of the following.....". Please explain ≤ 1 of the following. Is it any one of the following?

It should be"⩾1", From the following item in a, b, c.For a more accurate expression, it was modified to read as following:

"Sequelae were defined as consequent neurological developmental impairment (NDI) with at least one of the following : a.Bayley III cognitive, language, or motor composite scores <70 [8]; b: definitive cerebral palsy with any level of Gross Motor Function Classification System[9]; c: sensorineural or mixed hearing loss or unilatera l or bilateral visual impairment. "

10. Page 4, line 30-31, "gross motor function classification system level ≥ 1". Does it mean any level of GMFCS?

Yes. We revised as"definitive cerebral palsy with any level of Gross Motor Function Classification System"

10. Page 5, general characteristics section: If the children were enrolled at 18 m when the final diagnosis of sequelae was made, then keeping death and lost to follow-up as exclusion criteria will not be appropriate.

The time of enrollment was when the infant was diagnosed with BM, and I sincerely hope to keep death and lost to follow-up as exclusion criteria.

12. Table 1: there was a significant difference between the time the first USG was done from the onset of the disease in both the groups. Is there an explanation for this significant difference in the timing?

Because this study was a retrospective collection of ultrasound data, some infants were transferred from other hospitals and were not able to complete the cranial ultrasound in a timely manner; therefore, there was a significant difference between the time the first CUS, and in order to eliminate the possible bias in the results caused by this time difference, a stratified analysis of subgroups according to the time of CUS was performed as the acute phase(within 1 week) and the Post-acute phase(⩾1 week).

We have inserted appropriate explanations in this section of "Correlations between neuroimaging findings and BM sequelae" (Line 39-42)as follows:

"Because there was a significant difference between the time the first CUS, a stratified analysis of subgroups according to the time of CUS was performed as the Acute phase(within 1 week) and the Post-acute phase(⩾1 week)"

13. Table 2: in the row of meningeal thickening, the total N (%) should be 21 (20.5) instead of 14 (13.7).

I have checked the raw data and it is indeed a clerical error. I have corrected it in the table and in the corresponding part of the discussion section (Page 10, Line 19).

14. Although overall the acute abnormal CUS findings are not significantly different but individual findings like meningeal thickening, echogenic sulci, malacia lesion, IVH, ventricular dilatation were significantly different in the groups. This point can be highlighted in discussion or result section.

Relevant content is presented in the results section (Page 4,Line 42-46) as"CUS findings such as meningeal thickening, echogenic sulci, white matter hyperechogenicity (WMHE), changes in ventriculitis, intraventricular hemorrhage (IVH), and ventricular dilation were more likely to be found in the group with sequelae (all $p < 0.05$; Table 2). No independent risk factors for predicting neurological sequelae were identified by multivariate logistic regression analysis"

Given the word limit of the article, we have also partially presented in the discussion section, and based on the suggestions we have also made appropriate adjustments as follows：

"In our study meningeal thickening was statistically different only in the acute phase, which was mostly seen in the sequelae group.With very few cases with this sign in the acute phase, the result need to be explored further" (Page 9,Line 21)

"malacia lesion was more frequent in the group with sequelae both in the acute and post-acute phase($p < 0.05$),and totally sequelae developed in 10 (90.9%) of the 11 infants with leukomalacic lesions." (Page 9,Line 42-43)

"At the same time, ventriculitis was more commonly found in the group with sequelae than without in both the acute and post-acute phase($p < 0.05$)." (Page 10,Line 7)

14. Table 2: It is noticed that there are some findings on CUS done in the acute phase are significantly seen more in the sequelae group. On the other hand, a different set of findings in the post-acute CUS are associated with sequelae. This point should also be discussed.

The main different findings in the two phases are:

(1) Meningeal thickening was statistically different in the acute phase, which was mostly seen in the sequelae group, while was no statistically significant difference in the post-acute phase. The probable reason may be that this sign was found in very few cases in the acute phase, and only one case in the sequelae group, the results of which need to be explored in further research.Moreover, and the sign itself is not easy to be recognized by the screening process As mentioned later "Identifying such abnormalities is challenging, relying on the sonographer's experience and timing of the CUS. Despite their prevalence and value to assessing BM, these signs are temporary and unrelated to neurological outcomes. Consistently, we found that neither meningeal thickening nor echogenic sulci independently predicted neurological sequelae".(Page 9,Line 30-34)

(2) WMHE was statistically different only in the post-acute phase, we discuss as "This suggested that infants with BM and prolonged WMHE were prone to poor neurological outcomes."(Page 9,Line 50)

(3) IVH and Ventricular dilation were statistically different only in the acute phase.Significant ventricular dilatation early in the course of the disease is often indicative of rapid progression or large IVH that may be associated with the development of sequelae.

(4) EAFS enlargement was statistically different only in the post-acute phase.EAFS enlargement is generally not easily seen in the early stages of the disease and remains present in the later stages may be associated with poor prognosis.

Given the word limit of the article, We have added appropriate discussion as suggested：

“In our study meningeal thickening was statistically different only in the acute phase, which was mostly seen in the sequelae group.With very few cases with this sign in the acute phase, the result need to be explored further” (Page 9,Line 21)

“IVH and Ventricular dilation were more frequently found the sequelae group only in the acute phase,which suggested early ventricular dilatation might indicate a rapid progression or large IVH that may be associated with the poor outcome.”(Page 10,Line 14)

“which was statistically different only in the post-acute phase possible because it is generally not easily seen in the early stage of BM and remains present in the later stage may tend to poor prognosis.”(Page 10,Line 15)

Reply to Reviewer: 2

Dear Dr. Jaya Kaushik,

Thank you for reviewing our manuscript and for the constructive comments, which greatly helped us to improve the manuscript. We have revised the manuscript in accordance with the comments. And point-by-point responses to the comments were provided below.

We hope that the revision is acceptable, and your favorable consideration of our manuscript is greatly appreciated. Best regards！

1. the Best way to address this research question would have been a prospective cohort design where the sonographic findings were followed clinically for 1 year to determine the neurological outcome. The current retrospective chart review study design is an inferior design to address this question. Was it a retrospective chart review of medical records or a retrospective cohort? If it was a retrospective cohort, what was the age of children at the time of enrolment?

This study is a retrospective cohort study.The time of enrollment was when the infant was diagnosed with BM, and these infants were followed up regularly in our hospital after discharge.

The study is designed in 2023, Given the need for a minimum of 18 months of follow-up, the infants included were hospitalized before 31 December 2021 to ensure adequate follow-up time and outcome data available.

2. I believe only those who had BM in infancy were enrolled. However, there might be a variation in the age at presentation. I can see that infants were followed up till 18 months of age. In that case, BM should have occurred by 12 months of age. Hence, there will be a lot of

variation in the BM timing and follow-up duration. Were only cases of neonatal meningitis enrolled? Authors may clarify the methodology to avoid this confusion in readers' minds.

We included not only neonatal meningitis, but also infants aged <90 days with BM. The time of enrollment was when the infant was diagnosed with BM.

All infants with BM evaluated in our center are subjected to follow-up schedules， as they are considered to have a high risk in terms of neurodevelopmental problems. Professional physicians provide a thorough evaluation During the follow-up phase.

We judged it based on the outpatient information or the diagnostic information of Medical records at the time of a possible re-hospitalization due to illness. We consider infants with neurodevelopmental abnormalities above 18 months of age to have sequelae and be included in the sequelae group.

To prevent ambiguity, I modified the description of the summary section accordingly:

Abstract section：”Methods: We retrospectively assigned 132 infants diagnosed with BM from 2007 to 2021…………. (Page 2,Line 7)；Results: Overall, 102 infants with CUS and outcome data were recruited. 37/102 (36.3%) infants with neurological developmental impairments comprised the group with sequelae. 82.4%(84/102) of all infants had CUS neuroimaging abnormalities……..” (Page 2,Line 11)

Main text：This study conducted a retrospective analysis .The infants with CUS records and follow-up data for least 18 months were eventually included in the analysis. During the follow-up phase, certified examiners evaluated the infants using the Bayley Scales of Infant and Toddler Development III. (Page 3,Line 25)

3. The timing of the CUS needs clarification. Was it done within 7 days? Is there any timing with the lumbar puncture?

We have inserted appropriate explanations about The timing of the CUS in this section of “Correlations between neuroimaging findings and BM sequelae“ (Line 39-42)as follows:

“ Because there was a significant difference between the time the first CUS, a stratified analysis of subgroups according to the time of CUS was performed as the Acute phase(within 1 week) and the Post- acute phase(≥1 week)”

Unfortunately, we're not registered the time of lumbar punctures.

4. Was the developmental assessment done at 18 months of age? A single cut-off DQ<70 might be inappropriate to classify as sequelae. A reference to the same can be provided.

We consider infants with neurodevelopmental abnormalities above 18 months of age to have sequelae and be included in the sequelae group.We have made adjustments in the presentation based on the suggestions:

“Sequelae weredefined asconsequentneurological developmental impairment (NDI) at least one of the following: a: Bayley III cognitive, language, or motor composite scores <70 [8]; b: definitive cerebral palsy with any level of Gross Motor Function Classification System[9]; c: sensorineural or mixed hearing loss or

unilateral or bilateral visual impairment. All CUS procedures were conducted by experienced sonographers a ccording to neurosonography guidelines for infants[10,11].” (Page 3,Line 28)

5. Inclusion criteria mention raised CSF protein and low sugar without objectivity.

We have made adjustments in the presentation based on the suggestions:

The enrollment criteria were meet the following ① - ③or ④: ①temperature instabilities, lethargy, poor feeding, vomiting, convulsions, ②bulging anterior fontanelle, abnormal muscle tone, positive meningeal irritation sign; ③cerebrospinal fluid (CSF) white blood cell count (WBC) >20 $\times 10^6$/L, multinucleated cell ratio >0.6, increased total protein(>1.7 g/L for neonate and >0.4 g/L for infant aged 29-89 days), and decreased blood glucose(<2.2 mmol/L or CSF glucose/blood glucose ratio <0.4); ④positive bacteria in CSF smears or cultures.(Page 3,Line 13-18)

6. Were all children subjected to MRI as malformations are one of the exclusion criteria

Not all children in this study underwent MRI, and some were excluded for malformations based on CT, CUS or medical history.

7. Authors conclude that “careful CUS and monitoring of cerebral hemodynamics will prevent further cerebral insult” The statement may be reframed appropriately

We have made adjustments in the presentation as following:

“In conclusion, careful serial CUS imaging and monitoring of cerebral hemodynamic changes during the progression of BM are helpful with early adverse prognostic warnings.”(Page 11,Line 20-21)

VERSION 2 – REVIEW

**REVIEWER**	Dr. Ruchi Rai<br>Postgraduate Institute of Child Health<br>Sector 30, Noida<br>Noida<br>201303<br>India
**REVIEW RETURNED**	21-Jun-2024

**GENERAL COMMENTS**	I would like to make the following points regarding revision made by the authors.<br>Most points were addressed and clarified. Following points need attention:<br>1. It should be clearly mentioned in the topic as well as the methods section that it is a retrospective cohort study.

	2. As for the informed consent, the methods section mentions that informed consent was taken from parents or legal guardians. It should be mentioned that exemption from ethics committee was obtained for informed consent.<br>3. Regarding the criteria for diagnosis of bacterial meningitis, please clarify further whether it is either of the criteria i.e. either 1 or 2 or 3 or 4.

**REVIEWER**	Dr. Jaya Shankar Kaushik<br>All India Institute of Medical Sciences - Guwahati<br>Pediatrics<br>Department of Pediatrics, All India Institute of Medical Sciences, Guwahati, Assam, India<br>Guwahati<br>Assam<br>781101<br>India
**REVIEW RETURNED**	14-Jun-2024

**GENERAL COMMENTS**	None

VERSION 2 – AUTHOR RESPONSE

Dear Dr. Ruchi Rai,
Thank you for reviewing our revision and giving us the suggestion to improve the manuscript. We have revised the manuscript accordingly.Thanks again for your review comments. Best regards!

1. It should be clearly mentioned in the topic as well as the methods section that it is a retrospective cohort study.

Reply: I have revised the topic as "Prognostic value of cranial ultrasound findings in infants aged <90 days with bacterial meningitis: a single-center retrospective cohort study".
"retrospective cohort study" was mentioned in page 8 line 6,the part of "Study participants". Furthermore, we added in the part of "Methods" following the suggestion. Thanks!

2. As for the informed consent, the methods section mentions that informed consent was taken from parents or legal guardians. It should be mentioned that exemption from ethics committee was obtained for informed consent.

Reply:Yes, we've made changes based on the actual situation as "Exemption from ethics committee was obtained for informed consent mostly and a few participants who were less than 18 months of age at the time of their initial inclusion were informed the relevant content by telephone with their parents or legal guardians".

3. Regarding the criteria for diagnosis of bacterial meningitis, please clarify further whether it is either of the criteria i.e. either 1 or 2 or 3 or 4.
